# Comparative genome analysis reveals important genetic differences among serotype O1 and serotype O2 strains of *Y. ruckeri* and provides insights into host adaptation and virulence

**DOI:** 10.1002/mbo3.460

**Published:** 2017-03-20

**Authors:** Desirée Cascales, José A. Guijarro, Ana I. García‐Torrico, Jessica Méndez

**Affiliations:** ^1^ Área de Microbiología Departamento de Biología Funcional Facultad de Medicina IUBA Universidad de Oviedo Oviedo Spain

**Keywords:** adaptation, Genome, host, Serotype, Virulence, *Yersinia ruckeri*

## Abstract

Despite the existence of a commercial vaccine routinely used to protect salmonids against *Yersinia ruckeri*, outbreaks still occur, mainly caused by nonmotile and lipase‐negative strains (serotype O1 biotype 2). Moreover, epizootics caused by other uncommon serotypes have also been reported. At the moment, one of the main concerns for the aquaculture industry is the expanding range of hosts of this pathogen and the emergence of new biotypes and serotypes causing mortality in fish farms and against which the vaccine cannot protect. The comparative analysis of the genome sequences of five *Y. ruckeri* strains (150, CSF007‐82, ATCC29473, Big Creek 74, and SC09) isolated from different hosts and classified into different serotypes revealed important genetic differences between the genomes analyzed. Thus, a clear genetic differentiation was found between serotype O1 and O2 strains. The presence of 99 unique genes in Big Creek 74 and 261 in SC09 could explain the adaptation of these strains to salmon and catfish, respectively. Finally, the absence of 21 genes in ATCC29473 which are present in the other four virulent strains could underpin the attenuation described for this strain. The study reveals important genetic differences among the genomes analyzed. Further investigation of the genes highlighted in this study could provide insights into the understanding of the virulence and niche adaptive mechanisms of *Y. ruckeri*.

## 1 Background

*Yersinia ruckeri* is a gram‐negative rod‐shaped bacterium able to infect different fish species such as rainbow trout, carp, catfish, sturgeon, burbot, and perch. In salmonids, it causes enteric red mouth disease (ERM), a serious septicemic fish disease which is a major problem for aquaculture industries all over the world. Since its isolation in the United States in the 1950s (Ross, Rucker, & Ewing, 1966), the number of host species and the geographic distribution of this pathogen have increased considerably (Bastardo, Ravelo, & Romalde, 2015). Despite the existence of a reasonably effective immersion vaccine, outbreaks still occur, produced mainly by nonmotile and lipase‐negative strains (Arias et al., 2007; Austin, Robertson, & Austin, 2003; Calvez, Gantelet, Blanc, Douet, & Daniel, 2014; Fouz, Zarza, & Amaro, 2006).

In 1993, *Y. ruckeri* strains were classified into four serotypes with different subgroups (Romalde, Magariños, Barja, & Toranzo, 1993). The vast majority of epizootics in salmonid fish farms are caused by motile serotype O1, although epizootics caused by other uncommon serotypes have also been reported (Romalde, Planas, Sotelo, & Toranzo, 2003). Recently, a significant positive correlation between genetic and geographical distances was observed by Bastardo et al., 2015. Their results revealed that *Y. ruckeri* has experienced population changes that were probably induced by biogeography forces in the past and, much more recently, by adaptive processes resulting from aquaculture expansion.

During the last few years, nine genome sequences of *Y. ruckeri* strains, isolated from different niches have been uploaded onto NCBI (MKFJ00000000, NZ_CP011078, NZ_CP009539, JPFO00000000, CQBN00000000, CPUZ00000000, JPPT00000000, CCYO00000000, and JRWX00000000). Here, we present for the first time in this species a comparative analysis of five of those genomes belonging to strains isolated from different hosts and classified into different serotypes. The study reveals data that are important for a better understanding of the mechanisms underlying the niche adaptation and virulence of *Y. ruckeri*.

## 2 Materials and Methods

### 2.1 *Y. ruckeri* strains used for genome comparison

Five previously sequenced *Y. ruckeri* strains were selected for comparative genome analysis based upon their characteristics and hosts (Table 1). Three strains were from serotype O1, isolated from rainbow trout (*Oncorhynchus mykiss*), of which two were virulent (*Y. ruckeri* 150 and *Y. ruckeri* CSF007‐82), while the other, ATCC29473 type strain, was described as nonvirulent (Furones, Gilpin, Alderman, & Munn, 1990). The other two strains included in the analysis were *Y. ruckeri* Big Creek 74, belonging to serotype O2 and isolated from salmon, and *Y. ruckeri* SC09 isolated from catfish and of unknown serotype.

**Table 1:** List of *Y. ruckeri* strains used in comparative analysis

Characteristic	150	ATCC29473	CSF007‐82	Big Creek 74	SC09
Host	Rainbow trout	Rainbow trout	Rainbow trout	Chinook salmon	Catfish
Genome size (Mb)	3.82	3.77	3.83	3.69	3.92
Scaffolds	49	2	1	1	32
Contigs	169	15	1	1	32
GC (%)	46.8	47.4	47.5	47.6	47.45
CDS	3,538	3,377	3,530	3,136	3,651
RNAs	25	80	102	103	127
Serotype	O1	O1	O1	O2	–
Accession Number	MKFJ00000000	JPPT00000000	CCYO00000000	CP011078	JRWX00000000

### 2.2 Comparative analysis of *Y. ruckeri* genomes

Identification of putative protein‐encoding genes and annotation of *Y. ruckeri* genomes were performed with Rapid Annotation using Subsystem Technology (RAST) (Brettin et al., 2015). Before comparative analysis, the set of proteins from the five genomes selected were compared using BLAST to UniRef90 to associate each translation product to a Uniref90 protein. It was considered that a protein from one genome was orthologous to another one when they were in the same cluster. Based on this clusterization process, Venn diagrams were constructed with shared proteins (orthologous proteins) using the Venn diagram package in R (Chen & Boutros, 2011). Pairwise genome alignments were performed with MAUVE (Darling, Mau, Blattner, & Perna, 2004).

## 3 Results and Discussion

### 3.1 *Y. ruckeri* whole‐genome comparisons

**Figure 1:**
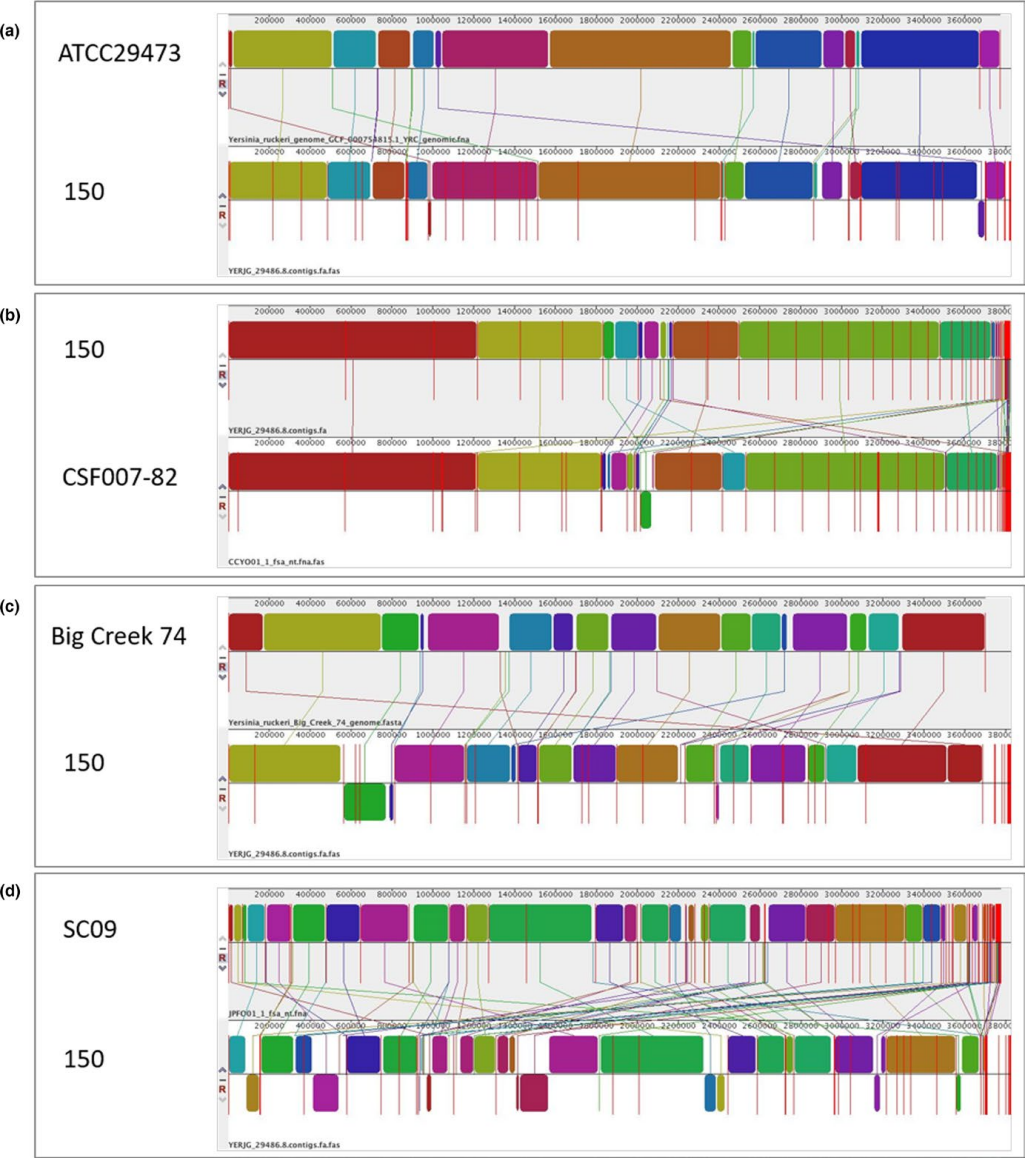
Chromosome alignments of *Y. ruckeri* 150 and (a) ATCC29473, (b) CSF007‐82, (c) Big Creek 74, and (d) SC09 using progressive Mauve. Local collinear blocks (LCBs) of conserved sequences among the strains are represented by rectangles of the same color. When these are above the chromosome (black line) they indicate the forward orientation and when they are positioned under the chromosome, the reverse orientation. Red lines represent contig limits. Connecting lines can be used to visualize genetic rearrangements

The pairwise full genome alignments revealed a mosaic pattern of homology organized in local collinear blocks (LCBs) between 150 and each of the other four strains (Figure 1). The 150 strain shares larger portions of genetic information with CSF007‐82 and ATCC24973, than it does with Big Creek74 and SC09. This result suggests that *Y. ruckeri* strains belonging to serotype O1 and having rainbow trout as a host (150, ATCC29473, CSF007‐82) are genetically more similar to each other than to other serotypes isolated from different animals, suggesting that differences in cell surface antigens and host specificity may have a markedly genetic base.

**Figure 2:**
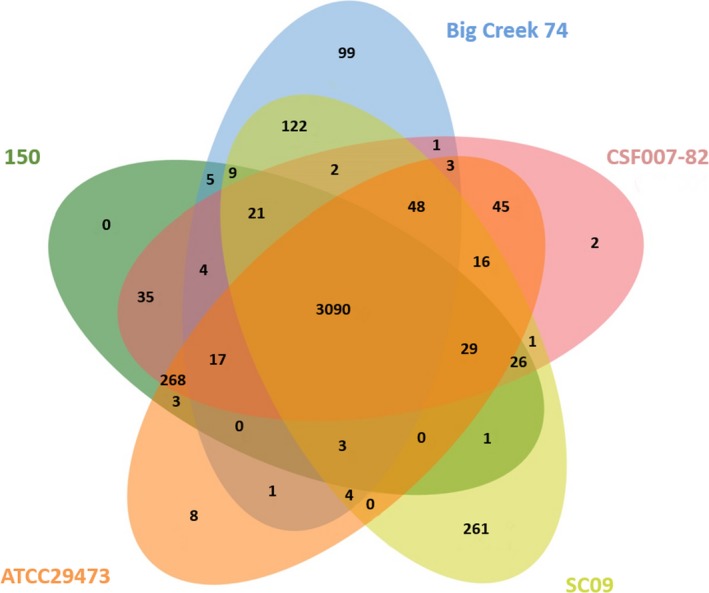
Venn diagram of *Y. ruckeri* 150, ATCC29473, CSF007‐82, Big Creek 74, and SC09. The number in the center of the diagram represents the genes shared by all species, whereas the digit on each branch indicates the number of unique or shared genes of the different strains

To identify orthologs shared by *Y. ruckeri* strains, a five‐way Venn diagram was made (Figure 2). The pangenome consists of 4,117 protein‐coding genes with a core of 3,090 genes (75.05%). A total of 370 genes were found to be strain‐specific, two genes corresponding to CSF007‐82, eight to ATCC29473, 99 to Big Creek 74, and 261 to SC09 genomes. Approximately, half of these unique genes (57%) were annotated as coding for hypothetical proteins. Interestingly, while serotype O1 strains isolated from rainbow trout have few unique genes (150 has none), the other two strains, Big Creek74 and SC09, have a great number, 99 and 261, respectively. Most of these genes could be related to host adaptation processes, in particular to survival in salmon in the first case and in catfish in the second.

As can be seen from the Venn diagram, the 150, ATCC29473, and CSF007‐82 strains share between them, and not with the other two strains, a total of 268 genes (6.5%). Similarly, the Big Creek 74 and SC09 strains share 122 (3%) genes which are absent in the genomes of serotype O1 strains. These data suggest a clear separation between serotype O1 strains and the Big Creek 74 and SC09 strains; so, it is tempting to speculate that SC09, whose serotype has not been described yet, could belong to serotype O2, as does Big Creek 74. This differentiation may constitute the genetic basis for the variation in serotype‐associated features among the strains.

### 3.2 Genes exclusively shared by serotype O1 strains

As mentioned above, a total of 268 genes were shared by 150, ATCC29473, and CSF007‐82 strains, all of them belonging to serotype O1 and isolated from rainbow trout. These genes include 113 which encode for hypothetical proteins, 33 are mobile genetic elements, 24 encode for phage‐related proteins, and 98 for proteins with different functions (Table 2). Thus, some of them are associated with restriction‐modification and toxin‐antitoxin systems. Both these systems have in common the death of cells that have lost one of the components (the antitoxin or the modification enzyme) and also their effect on global gene expression, which results in altered adaptive phenotypes. Thus, the antitoxin of the *Escherichia coli* MqsR–MqsA toxin‐antitoxin system directly represses the transcription of the gene encoding the master stress regulator RpoS, while the degradation of the antitoxin during stress leads to a switch from the high‐motility state to biofilm formation (Wang et al., 2011). In the same way, methylation events produced by restriction‐modification systems may affect nearby gene expression. Thus, methylation by Type III RM systems controls the expression of certain genes leading to two distinct cell types with two distinct phenotypes (“phasevarion”) (Srikhanta, Fox, & Jennings, 2010).

**Table 2:** Proteins exclusively shared by serotype O1 strains

Protein	150	ATCC2947	CSF007
Restriction‐modification systems			
Type I restriction‐modification system, specificity subunit S	BI323_00005	DJ39_RS07815	CSF007_RS15880
Eco57I restriction‐modification methylase family protein	BI323_06175	DJ39_RS04570	CSF007_RS05790
Type I restriction‐modification system, restriction subunit R	BI323_00080	DJ39_RS07890	CSF007_RS15805
Type I restriction‐modification system, DNA‐methyltransferase subunit M	BI323_00010	DJ39_RS07820	CSF007_RS15875
Restriction methylase	BI323_06275	DJ39_RS04465	CSF007_RS05895
Restriction methylase	BI323_07120	DJ39_RS07795	CSF007_RS06135
Antirestriction family protein	BI323_06250	DJ39_RS04490	CSF007_RS05870
Antirestriction family protein	BI323_000601	DJ39_RS07870	CSF007_RS15825
Toxin‐antitoxin systems			
YfjZ protein (Antitoxin to YpjF)	BI323_00070	DJ39_RS07880	CSF007_RS15815
Toxin YkfI	BI323_00075	DJ39_RS07885	CSF007_RS15810
Toxin_HigB‐2_	BI323_15350	DJ39_RS16625	CSF007_RS00015
Antitoxin ParD	BI323_16600	DJ39_RS11355	CSF007_RS09305
Toxin YkfI	BI323_06265	DJ39_RS04475	CSF007_RS05885
Legionaminic acid biosynthesis			
Dehydratase/C‐5‐epimerase	BI323_14300	DJ39_RS00115	CSF007_RS08290
Aminotransferase	BI323_14305	DJ39_RS00110	CSF007_RS08285
UDP‐N‐acetylglucosamine 2‐epimerase	BI323_14310	DJ39_RS00105	CSF007_RS08280
N‐acetylneuraminate synthase	BI323_14315	DJ39_RS00100	CSF007_RS08275
4‐amino‐6‐deoxy‐N‐Acetyl‐D‐hexosaminyl‐(Lipid carrier) acetyltrasferase	BI323_14320	DJ39_RS00095	CSF007_RS08270
Mannose‐1‐phosphate guanyltransferase	BI323_14325	DJ39_RS00090	CSF007_RS08265
Oxidoreductase, NAD‐binding Rossmann fold family protein	BI323_14330	DJ39_RS00085	CSF007_RS08260
Acylneuraminate cytidylyltransferase	BI323_14335	DJ39_RS00080	CSF007_RS08255
Dehydrogenase	BI323_14340	DJ39_RS00075	CSF007_RS08250
Polysaccharide biosynthesis family protein	BI323_14345	DJ39_RS00070	CSF007_RS08245
Aminotransferase	BI323_14360	DJ39_RS00055	CSF007_RS08230
Imidazole glycerol phosphate synthase subunit HisH	BI323_14365	DJ39_RS00050	CSF007_RS08225
Imidazole glycerol phosphate synthase	BI323_14370	DJ39_RS00045	CSF007_RS08220
Epimerase/dehydratase	BI323_14375	DJ39_RS00040	CSF007_RS08215
UDP‐2‐acetamido‐2,6‐dideoxy‐beta‐L‐talose‐4‐dehy drogenase	BI323_14380	DJ39_RS00035	CSF007_RS08210
UDP‐N‐acetylglucosamine 2‐epimerase	BI323_14385	DJ39_RS00030	CSF007_RS08205
Glycosyl transferases group 1 family protein	BI323_14390	DJ39_RS00025	CSF007_RS08200
Tryptophan synthase beta chain like	BI323_14395	DJ39_RS00020	CSF007_RS08195
DNA repair			
DNA repair RadC family protein	BI323_00065	DJ39_RS07875	CSF007_RS15820
ATPase involved in DNA repair	BI323_15390	DJ39_RS16660	CSF007_RS00050
DNA_repair_ATPase_	BI323_06180	DJ39_RS04565	CSF007_RS05795
ATPase involved in DNA repair	BI323_06215	DJ39_RS04530	CSF007_RS05830
RadC family DNA repair protein	BI323_06255	DJ39_RS04485	CSF007_RS05875
Transcriptional regulators			
Regulator	BI323_01350	DJ39_RS09205	CSF007_RS01385
XRE_family_transcriptional_regulator	BI323_15355	DJ39_RS16630	CSF007_RS00020
Transcription_factor	BI323_16050	DJ39_RS16900	CSF007_RS16935
XRE_family_transcriptional_regulator	BI323_05810	DJ39_RS16260	CSF007_RS05430
Type IV secretion system			
Type IV secretion‐system coupling DNA‐binding domain protein	BI323_15400	DJ39_RS16670	CSF007_RS00060
TrbA	BI323_15410	DJ39_RS16680	CSF007_RS00070
RelB/StbD replicon stabilization protein (Antitoxin to RelE/StbE)	BI323_15420	DJ39_RS16690	CSF007_RS00080
Conjugal transfer/type IV secretion DotA/TraY family protein	BI323_15425	DJ39_RS16680	CSF007_RS00085
IncI1 plasmid conjugative transfer protein TraW	BI323_15435	DJ39_RS16705	CSF007_RS00095
IncI1 plasmid conjugative transfer protein TraU	BI323_15440	DJ39_RS16710	CSF007_RS00100
IncI1 plasmid conjugative transfer protein	BI323_15455	DJ39_RS16725	CSF007_RS00115
IncI1 plasmid conjugative transfer protein TraO	BI323_15465	DJ39_RS16735	CSF007_RS00125
TraN	BI323_15470	DJ39_RS16740	CSF007_RS00130
TraM	BI323_15475	DJ39_RS16745	CSF007_RS00135
TraC	BI323_15485	DJ39_RS16755	CSF007_RS00145
TraK	BI323_15490	DJ39_RS16760	CSF007_RS00150
Plasmid transfer ATPase TraJ	BI323_15495	DJ39_RS16765	CSF007_RS00155
TraI	BI323_15500	DJ39_RS16770	CSF007_RS00160
IncI1 plasmid conjugative transfer protein TraH	BI323_15505	DJ39_RS16775	CSF007_RS00165
Prepilin	BI323_15525	DJ39_RS16795	CSF007_RS00185
General_secretion_pathway_protein_GspF	BI323_15530	DJ39_RS16800	CSF007_RS00190
PilO	BI323_15545	DJ39_RS16815	CSF007_RS00205
IncI1 plasmid conjugative transfer lipoprotein PilN	BI323_15550	DJ39_RS16820	CSF007_RS00210
Type IVB pilus formation outer membrane protein, R64 PilN family	BI323_15555	DJ39_RS16825	CSF007_RS00215
Transferases			
Methyltransferase domain protein	BI323_16780	DJ39_RS04020	CSF007_RS16255
Glycosyl transferase, family 2	BI323_06290	DJ39_RS04450	CSF007_RS05910
Other proteins			
ATP/GTP‐binding_protein	BI323_00035	DJ39_RS07845	CSF007_RS15850
Bipolar DNA helicase HerA	BI323_10945	DJ39_RS01005	CSF007_RS11255
Phosphopantetheine attachment site family protein	BI323_13885	DJ39_RS06465	CSF007_RS15645
AMP‐dependent synthetase	BI323_13890	DJ39_RS06470	CSF007_RS15640
3‐oxoacyl‐[acyl‐carrier protein] reductase	BI323_13895	DJ39_RS06475	CSF007_RS15635
Polysaccharide deacetylase	BI323_13905	DJ39_RS06485	CSF007_RS15625
Endonuclease	BI323_15395	DJ39_RS16665	CSF007_RS00055
Putative ATP‐binding protein involved in virulence	BI323_16055	DJ39_RS16905	CSF007_RS16930
ATP‐dependent DNA helicase RecG	BI323_16060	DJ39_RS16910	CSF007_RS16925
Chromosome segregation ATPase	BI323_16110	DJ39_RS16960	CSF007_RS16875
Initiator Replication family protein	BI323_16595	DJ39_RS11350	CSF007_RS09310
Plasmid_stabilization_protein_	BI323_16605	DJ39_RS11360	CSF007_RS09300
Cobyrinic acid a,c‐diamide synthase	BI323_16640	DJ39_RS11395	CSF007_RS09265
Glycosaminoglycan attachment site	BI323_16685	DJ39_RS14755	CSF007_RS03940
Retron‐type RNA‐directed DNA polymerase	BI323_16690	DJ39_RS14760	CSF007_RS03935
Sulfur transport family protein	BI323_16730	DJ39_RS00135	CSF007_RS17245
Cytotoxic family protein	BI323_16760	DJ39_RS17105	CSF007_RS17365
Low calcium response locus protein T	BI323_16800	DJ39_RS04040	CSF007_RS16275
ParB/RepB/Spo0J family partition domain protein	BI323_16820	DJ39_RS16600	CSF007_RS16685
Plasmid partition protein A	BI323_16825	DJ39_RS16605	CSF007_RS16690
ATP‐dependent Lon protease	BI323_06155	DJ39_RS04590	CSF007_RS05770
alkaline_phosphatase	BI323_06160	DJ39_RS04585	CSF007_RS05775
ABC_transporter_ATP‐binding_protein_	BI323_06170	DJ39_RS04575	CSF007_RS05785
DNA‐binding_protein_	BI323_06200	DJ39_RS04545	CSF007_RS05815
AlpA family protein	BI323_06210	DJ39_RS04535	CSF007_RS05825
GTPase	BI323_06225	DJ39_RS04520	CSF007_RS05840
YagBYeeUYfjZ family protein	BI323_06270	DJ39_RS04470	CSF007_RS05890
DNA‐binding protein	BI323_06285	DJ39_RS04455	CSF007_RS05905
37‐kD nucleoid‐associated bacterial protein	BI323_06340	DJ39_RS00940	CSF007_RS06920
(p)ppGpp_synthetase_	BI323_06345	DJ39_RS00935	CSF007_RS06915
Colicin‐Ib	BI323_06365	DJ39_RS17065	CSF007_RS17470
AAA ATPase	BI323_06375	DJ39_RS00905	CSF007_RS06885
AAA ATPase	BI323_06400	DJ39_RS00875	CSF007_RS06855
Invasin	BI323_06460	DJ39_RS17045	CSF007_RS17450
DNA‐directed RNA polymerase subunit sigma70	BI323_09490	DJ39_RS13885	CSF007_RS05930
ATP‐binding_protein_	BI323_09540	DJ39_RS13835	CSF007_RS05980

A relevant finding shared by all serotype O1 strains was related to a cluster of genes which are involved in the biosynthesis of the legionaminic acid, a nine‐carbon diamino monosaccharide that is found coating the surface of various bacterial human pathogens, being the major component of the LPS. Interestingly, these genes which are grouped in a cluster of at least 18 genes are absent in other *Yersinia* species but present in other aquatic bacteria such as *Vibrio vulnificus*,* Aeromonas salmonicida*,* Vibrio fischeri*, or *Photobacterium profundum*. It is possible that this cluster provides an adaptive advantage for surviving in the aquatic environment or, as happens in some organisms such as *Campylobacter jejuni* (Zebian et al., 2016), it is related to virulence. This is because legionaminic acid is essential for flagella assembly in several species (Morrison & Imperiali, 2014) and for this reason, the genes involved in its biosynthesis are novel targets for the development of antivirulence agents (Table 2).

Other genes which are exclusive to O1 serotype strains code for a bacteriocin similar to colicin‐Ib of *Escherichia coli* (WP_062877260) and virulence factors such as a type IV secretion system previously analyzed by Méndez et al., (2009) and an invasin present in other enterobacteriaceae such as *Yersinia pestis* (EIR59646), *Y. pseudotuberculosis* (WP_050128752), and *Edwarsiella tarda* (WP_047059316) (Table 2).

### 3.3 Genes exclusively shared by Big Creek 74 and SC09 strains

**Figure 3:**
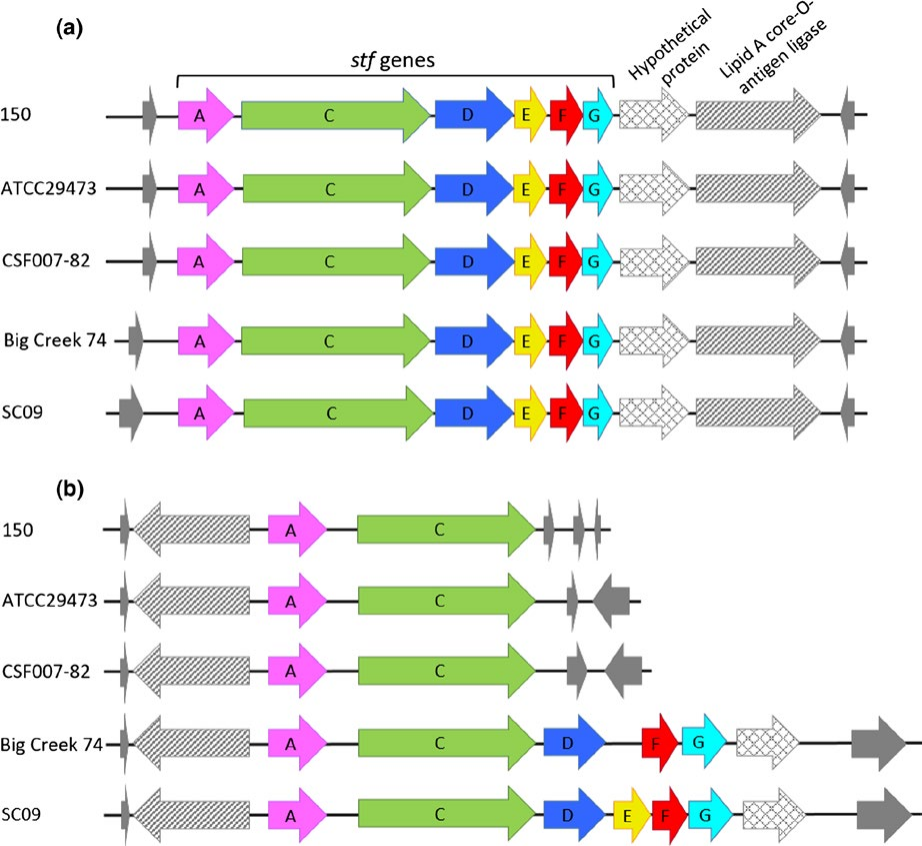
Analysis of the *stf* genes in *Y. ruckeri* genomes. Two copies of the *stf* cluster were found in *Y. ruckeri* strains. One copy of the cluster *stfACDEFG* is complete in the five strains (a), while a second copy is only complete in SC09, and with the exception of *stfE* gene, in Big Creek 74. The second copy of the *stf* cluster in 150, ATCC29473, and CSF007‐82 strains is only constituted by *stfA* and *stfC* genes (b). Note that the gene represented by a striped arrow, which encodes a lipid A core‐O‐antigen ligase, was affected by a translocation and an inversion event, resulting in a different localization in the two clusters

As indicated in the Venn diagram (Figure 2), Big Creek 74 and SC09 share a total of 122 genes which include 37 ORFs encoding for proteins of unknown function, 50 for phage‐related proteins, and 35 encode for proteins with similarity to proteins involved in a variety of functions such as restriction‐modification systems, toxin‐antitoxin systems or proteins involved in fimbriae synthesis (Table 3). One such case is that of a cluster involved in fimbriae biosynthesis, similar to the *Stf* cluster of *Salmonella typhimurium* which has been associated with differences in virulence and host range between the different serotypes (Emmerth, Goebel, Miller, & Hueck, 1999). Although one *Stf* cluster copy is present in the five genomes analyzed (Figure 3a), an additional complete copy of this cluster was only found in the genome of SC09 and, with the exception of the gene encoding the minor fimbriae subunit (*stfE*), also in Big Creek 74 (Figure 3b). The last copy seems to be the result of several genetic rearrangements so it is probably not functional in those strains (Figure 3b).

**Table 3:** Proteins shared by Big Creek 74 and SC09 strains

Protein	SC09	Big creek 74
Restriction‐modification system		
TypeI R‐M System, specificity subunitS	NJ56_RS02590	UGYR_RS06280
Toxin‐antitoxin system		
Antitoxin to RelE/StbE	NJ56_RS02580	UGYR_RS12460
Replicon stabilization toxin RelE	NJ56_RS02585	UGYR_RS12465
Fimbria		
Fimbrial protein StfD	NJ56_RS14520	UGYR_RS0378
Exotoxin/MrfF	NJ56_RS14525	UGYR_RS03790
Putative fimbrial membrane protein	NJ56_RS14530	UGYR_RS03795
Exotoxin	NJ56_RS14540	UGYR_RS03805
Fimbrial anchoring protein FimD	NJ56_RS10070	UGYR_RS06285
Fimbrial subunit	NJ56_RS17965	UGYR_RS16650
Exotoxin/minnor fimbrial subunit	NJ56_RS14535	UGYR_RS03800
Fimbria‐like adhesine SfmA	NJ56_RS16255	UGYR_RS05635
Fimbrial periplasmic chaperone SfmC	NJ56_RS10065	UGYR_RS06280
Fimbrial like adhesine prot	NJ56_RS17890	UGYR_RS16665
PilN family type IV pilus biogenesis protein	NJ56_RS10080	UGYR_RS06295
GCN5‐related N‐acetyltransferase	NJ56_RS10095	UGYR_RS06310
Insecticidal virulence protein		
Probable insecticidal protein	NJ56_RS10100	UGYR_RS06315
Putative toxin subunit	NJ56_RS10110	UGYR_RS06325
Putative insecticidal toxin complex	NJ56_RS10115	UGYR_RS06330
Sorbitol sorbose utilization		
Arabinose 5‐phosphate isomerase	NJ56_RS11170	UGYR_RS07380
Transcriptional regulator/glucitol operon activator prot	NJ56_RS11160	UGYR_RS07370
Transcriptional repressor of fructose operon DeoR family	NJ56_RS11165	UGYR_RS07375
Sorbitol‐6‐phosphate 2‐dehydrogenase	NJ56_RS11155	UGYR_RS07365
PTS glucitol/sorbitol transporter subunit IIB	NJ56_RS11145	UGYR_RS07355
PTS glucitol/sorbitol transporter subunit IIA	NJ56_RS11150	UGYR_RS07360
PTS glucitol/sorbitol transporter subunit IIC	NJ56_RS11140	UGYR_RS07350
Other proteins		
Transcriptional regulator LysR family	NJ56_RS10085	UGYR_RS06300
S‐adenosylhomocysteine hydrolase	NJ56_RS02625	UGYR_RS12500
rimosomal protein	NJ56_RS14055	UGYR_RS02835
Outer memb component of tripartite multidrug resistance system	NJ56_RS10260	UGYR_RS06475
Chromosome partitioning protein ParA	NJ56_RS11555	UGYR_RS07765
Tfp pilus assembly protein%2C major pilin PilA	NJ56_RS09235	UGYR_RS02515
yclopropane‐fatty‐acyl‐phospholipid synthase	NJ56_RS11560	UGYR_RS07770
FAD‐dependent oxidoreductase	NJ56_RS11550	UGYR_RS07760
Inorganic pyrophosphatase/exopoliphosphatase	NJ56_RS02630	UGYR_RS12505
Spermidine/putrescine ABC trasporter permease	NJ56_RS06425	UGYR_RS16290

Three insecticidal toxin complexes (tc)‐like proteins were also identified as unique in these strains. They are similar to the TcdA, TcdB, and TcdC proteins of *Vibrio parahaemolyticus,* which are involved in the production of acute hepatopancreatic necrosis disease in penaeid shrimp (Tang & Lightner, 2014).

**Figure 4:**
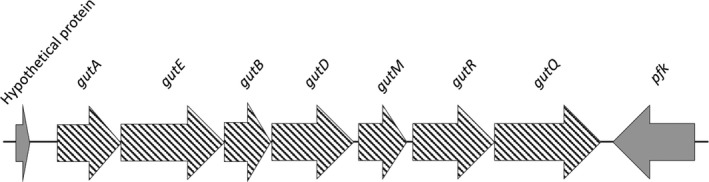
Genetic organization of the *gut* operon in *Y. ruckeri* Big Creek 74 and SC09 strains. *gutA, gutE*, and *gutB:* subunits of a glucitol/sorbitol‐specific transporter, *gutD*: sorbitol‐6‐phosphate 2‐dehydrogenase, *gutM*: glucitol operon activator protein, *gutR*: glucitol operon repressor protein, *gutQ*: an arabinose 5‐phosphate isomerase. *pfk* encodes for a 6‐phosphofructokinase class II

One of the most interesting findings was that Big Creek 74 and SC09 strains share a cluster of seven genes involved in the utilization of sorbitol (Figure 4), a previously described characteristic associated with *Y. ruckeri* serotype O2 strains (Davies & Frerichs, 1989), which supports the hypothesis that SC09 belongs to this serotype. The genes are similar to *gutAEBD* of *E. coli* (Yamada & Saier, 1987). The first three genes encode the three subunits of the sorbitol transporter of the phosphoenolpyruvate‐dependent phosphotransferase system (PTS), involved in the uptake and phosphorylation of sorbitol, while *gutD* encodes a sorbitol‐6‐phosphate 2‐dehydrogenase that synthesizes D‐fructose 6‐phosphate from D‐sorbitol 6‐phosphate. In *Y. ruckeri* strains, as occurs in *E. coli*, downstream of *gutAEBD* genes, there are two transcriptional regulators, an activator and a repressor of the sorbitol operon, similar to *gutM* and *gutR* of *E. coli*, respectively. Downstream of *gutR*, is located *gutQ*, which encodes an arabinose 5‐phosphate isomerase involved in LPS biosynthesis. Although the role of this protein in the sorbitol metabolism is unclear, it could be a regulatory molecule involved in expression of the *gut* operon (Meredith & Woodard, 2005). In the plant pathogen *Erwinia amylovora*, the presence of this operon has been linked to virulence and suggested to contribute to host specificity (Aldridge, Metzger, & Geider, 1997).

### 3.4 Unique genes of Big Creek 74

As was seen in the Venn diagram (Figure 2), Big Creek 74 strain has a total of 99 unique genes, which include 53 encoding hypothetical proteins, eight phage genes, four mobile genetic elements, and 34 genes which encode proteins with known function. As was mentioned above, the presence of some of these genes may underpin its adaptation to salmon, since the host of the other four strains is rainbow trout or catfish.

Among the proteins with known function (Table S1), we can find restriction‐modification systems, transcriptional regulators, transferases, or proteins involved in polysaccharide biosynthesis. Especially interesting is the gene encoding an ATP‐dependent Clp protease proteolytic subunit, a relevant regulatory enzyme in different bacteria, related also to virulence, environmental adaptation, and antibiotic resistance in microorganisms such as *Staphylococcus aureus* (Frees, Gerth, & Ingmer, 2014) or the fish pathogen *Pseudomonas fluorescens* (Liu, Chi, & Sun, 2015).

### 3.5 Unique genes of SC09

SC09 has a total of 261 genes that are not present in the other strains, 148 of them encode hypothetical proteins, 17 are phage‐related genes, nine mobile genetic elements, and the rest encode proteins with different functions. As was suggested for Big Creek 74, some of these genes may underpin the adaptation of this strain to survive inside the host (catfish) or under certain environmental conditions. Among these unique proteins are transcriptional regulators, proteins related to type IV secretion systems, restriction‐modification, and toxin‐antitoxin components and proteins associated with cellular energy homeostasis (Table S2). One of the most interesting proteins is a thymidylate synthase, an enzyme linked to virulence in several microorganisms such as *Staphylococcus aureus* (Kriegeskorte et al., 2014) or *Salmonella typhimurium*, in which it was necessary for intracellular growth, both in macrophage‐like and Hep‐2 human epithelial cell lines (1) and also for complete virulence in a BALB/c mice model (Kok, Bühlmann, & Pechère, 2001).

A finding which is worthy of further investigation was the presence, only in this strain, of a cluster of 12 genes related to cell wall polysaccharide biosynthesis, in particular the O‐antigen.

### 3.6 Genes solely absent in the avirulent strain ATCC29473

**Figure 5:**
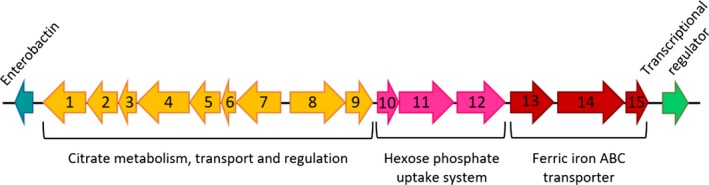
Genetic organization of the DNA region absent in ATCC29473 and present in the other strains. The region contains genes encoding for an enterobactin‐like siderophore (blue), nine genes involved in the uptake and metabolism of citrate (yellow), a group of three genes related to hexose phosphate uptake (pink), three genes involved in iron transport (red), and a Crp‐Fnr family transcriptional regulator (green). 1: Citrate succinate antiporter, 2: 2‐(5′’‐triphosphoribosyl)‐3′‐dephosphocoenzyme‐A synthase, 3: Apo‐citrate lyase phosphoribosyl‐dephospho‐CoA transferase, 4: Citrate lyase alpha chain, 5: Citrate lyase beta chain, 6: Citrate lyase gamma chain acyl carrier protein, 7: [Citrate [pro‐3S]‐lyase] ligase, 8: Sensor kinase, 9: Transcriptional regulatory protein, 10: Transcriptional regulatory protein, 11: Sensor histidine protein kinase glucose‐6‐phosphate specific, 12: Hexose phosphate uptake regulatory protein, 13: Ferric iron ABC transporter iron‐binding protein, 14: Ferric iron ABC transporter permease protein, 15: Ferric iron ABC transporter binding subunit

Among the five strains included in the study, ATCC29473 was defined as avirulent. In this sense, it was intriguing to analyze which genes are absent in this strain and present in the others, in order to elucidate the genetic basis of its attenuation. A total of 21 genes were found (Table 4), all of them encoding proteins with an assigned function, which were probably lost during the evolution of this strain. It is significant that 17 out of 21 genes are adjacent in the other four genomes from virulent strains (Figure 5). This region of 19,566 bp contains genes encoding for a Crp‐Fnr family transcriptional regulator, a hypothetical protein, an enzyme related to an enterobactin‐like siderophore and three different gene clusters: one formed by three genes involved in iron transport, a group of three genes related to hexose phosphate uptake; and a region containing nine genes involved in the uptake and metabolism of citrate. Since most of these genes are related to virulence (Gray, Freitag, & Boor, 2006; Moisi et al., 2013; Urbany & Neuhaus, 2008), it is possible that the absence of this region could explain, in some way, the attenuation of *Y. ruckeri* ATCC29473. This is important for future studies and may help to shed light on the virulence of the species.

**Table 4:** Proteins absent in the avirulent strain ATCC29473 and present in the other strains

Protein	150	BIG CREEK 74	CSF007‐82	SC09
Aromatic amino acid decarboxylase	BI323_03940	UGYR_RS01810	CSF007_RS12055	NJ56_RS08540
Serine hydrolase family protein	BI323_03955	UGYR_RS01805	CSF007_RS12040	NJ56_RS08535
Virulence factor	BI323_03960	UGYR_RS01800	CSF007_RS12035	NJ56_RS08530
Crp‐like helix‐turn‐helix domain protein	BI323_16505	UGYR_RS04050	CSF007_RS14555	NJ56_RS14785
Fe(3 + ) ions import ATP‐binding protein FbpC	BI323_16510	UGYR_RS04045	CSF007_RS14560	NJ56_RS14780
Putative binding protein‐dependent transport system%2C inner‐membrane component	BI323_16515	UGYR_RS04040	CSF007_RS14565	NJ56_RS14775
Bacterial extracellular solute‐binding family protein	BI323_16520	UGYR_RS04035	CSF007_RS14570	NJ56_RS14770
Major Facilitator Superfamily protein	BI323_16525	UGYR_RS04030	CSF007_RS14575	NJ56_RS14765
Sensor histidine protein kinase UhpB, glucose‐6‐phosphate specific	BI323_16530	UGYR_RS04025	CSF007_RS14580	NJ56_RS14760
Bacterial regulatory s, luxR family protein	BI323_16535	UGYR_RS04020	CSF007_RS14585	NJ56_RS14755
Transcriptional regulatory protein	BI323_16540	UGYR_RS04015	CSF007_RS14590	NJ56_RS14750
Sensor histidine kinase DpiB	BI323_16545	UGYR_RS04010	CSF007_RS14595	NJ56_RS14745
[citrate (Pro‐3S)‐lyase] ligase	BI323_16550	UGYR_RS04005	CSF007_RS14600	NJ56_RS14740
Citrate lyase acyl carrier protein	BI323_16555	UGYR_RS04000	CSF007_RS14605	NJ56_RS14735
Citrate lyase subunit beta	BI323_16560	UGYR_RS03995	CSF007_RS14610	NJ56_RS14730
Citrate lyase alpha chain	BI323_16565	UGYR_RS03990	CSF007_RS14615	NJ56_RS14725
Holo‐ACP synthase CitX	BI323_16570	UGYR_RS03985	CSF007_RS14620	NJ56_RS14720
Probable 2‐(5’’‐triphosphoribosyl)‐3′‐dephosphocoenzyme‐A synthase	BI323_16575	UGYR_RS03980	CSF007_RS14625	NJ56_RS14715
Citrate carrier	BI323_16580	UGYR_RS03975	CSF007_RS14630	NJ56_RS14710
4′‐phosphopantetheinyl transferase siderophore	BI323_16585	UGYR_RS03970	CSF007_RS14635	NJ56_RS14705
Holin	BI323_09445	UGYR_RS04445	CSF007_RS17025	NJ56_RS17590

## 4 Conclusion

In this study, is presented for the first time, the comparative analysis of five genome sequences of *Y. ruckeri*. Although the five strains shared approximately 75% of their genes, our study has revealed important genetic differences between the five genomes. Aside from the genetic differentiation found between serotype O1 and O2 strains, especially relevant are the high number of unique genes found in Big Creek 74 and SC09 in relation to serotype O1 strains and the 21 genes absent in the avirulent strain ATCC29473. These findings could explain the host specificity of the first two strains or the virulence attenuation of ATCC29473. Further investigation of those genes will provide insights into understanding the pathogenesis and the adaptive mechanisms to different environments of *Y. ruckeri*.

## Conflict of Interests

The authors declare no competing interests.

## Ethics Statement

This research did not involve any human or animal subjects, materials, or data and therefore did not require any ethics oversight or approval in these respects.
