# Supplementary material for: Comparative genome analysis reveals important genetic differences among serotype O1 and serotype O2 strains of Y. ruckeri and provides insights into host adaptation and virulence
Source: Microbiologyopen. 2017 Mar 20;6(4):e00460. doi: 10.1002/mbo3.460 (PMC5552943; doi:10.1002/mbo3.460)
Supplement: Supplementary file 1 [file MBO3-6-na-s001.docx]

**Table S1. Exclusive proteins of Big Creek 74**

| **Protein** | **Locus** | |
| --- | --- | --- |
| **Restriction-Modification systems** |  |  |
| Restriction endonuclease | UGYR_RS12300 |  |
| Type I restriction enzyme EcoKI M protein | UGYR_RS12280 |  |
| Restriction endonuclease | UGYR_RS12305 |  |
| Specificity determinant for hsdM and hsdR | UGYR_RS12285 |  |
| **Transcritional regulators** |  |  |
| Repressor | UGYR_RS02755 |  |
| Rha family transcriptional regulator | UGYR_RS04430 |  |
| Regulatory protein | UGYR_RS04515 |  |
| Repressor | UGYR_RS04520 |  |
| **Polysaccharide biosynthesis** |  |  |
| dTDP-4-dehydrorhamnose reductase | UGYR_RS14790 |  |
| Polysaccharide biosynthesis family protein | UGYR_RS13615 |  |
| UDP-N-acetylglucosamine 2-epimerase | UGYR_RS14785 |  |
| Polysaccharide pyruvyl transferase family protein | UGYR_RS13605 |  |
| **Transferases** |  |  |
| Glycosyl transferase | UGYR_RS14780 |  |
| Glycosyl transferases group 1 family protein | UGYR_RS13600 |  |
| Glycosyl transferases group 1 family protein | UGYR_RS14800 |  |
| Methyltransferase small domain protein | UGYR_RS14770 |  |
| Acyltransferase family protein n= | UGYR_RS13625 |  |
| Cytosine-specific methyltransferase | UGYR_RS12420 |  |
| **Other proteins** |  |  |
| Replication protein B | UGYR_RS02770 |  |
| Helicase UvrD | UGYR_RS06215 |  |
| Helicase | UGYR_RS06255 |  |
| DEAD/DEAH box helicase | UGYR_RS12290 |  |
| DEAD/DEAH box helicase | UGYR_RS12275 |  |
| Chromosome segregation protein SMC | UGYR_RS06220 |  |
| Peptidase M15 | UGYR_RS04440 |  |
| Endopeptidase | UGYR_RS04435 |  |
| ATP-dependent Clp protease proteolytic subunit | UGYR_RS04405 |  |
| Dipicolinate synthase | UGYR_RS12380 |  |
| Cupin fold metallo, WbuC family protein | UGYR_RS14775 |  |
| Ornithine decarboxylase (Fragment) | UGYR_RS12310 |  |
| Nitroreductase family protein | UGYR_RS13610 |  |
| Toxin | UGYR_RS06225 |  |
| Histidine kinase | UGYR_RS12425 |  |
| DNA-binding protein | UGYR_RS04510 |  |
