# Supplementary material for: Comparative genome analysis reveals important genetic differences among serotype O1 and serotype O2 strains of Y. ruckeri and provides insights into host adaptation and virulence
Source: Microbiologyopen. 2017 Mar 20;6(4):e00460. doi: 10.1002/mbo3.460 (PMC5552943; doi:10.1002/mbo3.460)
Supplement: Supplementary file 2 [file MBO3-6-na-s002.docx]

**Table S2. Exclusive proteins of SCO9**

| **Protein** | **Locus** |
| --- | --- |
| **Conjugal transfer proteins** |  |
| Conjugative coupling factor TraD | NJ56_RS01525 |
| Conjugal transfer protein TraD | NJ56_RS17275 |
| Conjugal transfer protein TraN | NJ56_RS17305 |
| Conjugal transfer protein TraB | NJ56_RS17340 |
| Conjugal transfer protein TraB | NJ56_RS17100 |
| Conjugal transfer protein TraH | NJ56_RS17290 |
| Conjugal transfer protein TraW | NJ56_RS17320 |
| Conjugal transfer protein TraL | NJ56_RS17355 |
| Conjugal transfer protein TraU | NJ56_RS17315 |
| Protein TraE | NJ56_RS17350 |
| Conjugal transfer protein TraI | NJ56_RS17270 |
| Conjugal transfer protein TrbB | NJ56_RS17295 |
| Conjugal transfer protein TraC | NJ56_RS17330 |
| Conjugal transfer protein TraF | NJ56_RS17300 |
| Conjugal transfer protein | NJ56_RS01515 |
| Conjugal transfer protein | NJ56_RS01570 |
| Conjugal transfer protein TrbI | NJ56_RS01575 |
| Conjugal transfer protein | NJ56_RS01615 |
| Conjugal transfer protein | NJ56_RS01550 |
| Conjugal tranfer protein | NJ56_RS01610 |
| Conjugal transfer protein | NJ56_RS01475 |
| Conjugal transfer protein | NJ56_RS01605 |
| IncF plasmid conjugative transfer protein TraN | NJ56_RS17990 |
| Conjugal pilus assembly protein TraF | NJ56_RS16815 |
| Sex pilus assembly and mating pair formation protein TrbC | NJ56_RS17065 |
| Conjugal transfer pilus assembly protein TraU | NJ56_RS17070 |
| Conjugal transfer pilus assembly protein TraH | NJ56_RS17045 |
| Conjugal transfer ATP-binding protein TraC | NJ56_RS17090 |
| TraK protein | NJ56_RS17105 |
| Conjugal transfer protein | NJ56_RS01580 |
| IncF plasmid conjugative transfer protein TraN | NJ56_RS17085 |
| Type IV conjugative transfer system protein TraV | NJ56_RS17095 |
| **Restriction-modification** |  |
| Restriction methylase | NJ56_RS17400 |
| Methyltransferase | NJ56_RS17575 |
| Cytosine-specific methyltransferase | NJ56_RS02490 |
| Antirestriction ArdB family protein | NJ56_RS01655 |
| Antirestriction protein | NJ56_RS01675 |
| Restriction endonuclease | NJ56_RS02485 |
| Type II restriction endonuclease | NJ56_RS02500 |
| **Toxin-antitoxin systems** |  |
| Addiction module antitoxin | NJ56_RS16010 |
| RelE toxin protein | NJ56_RS17395 |
| **DNA interaction** |  |
| DNA-binding protein | NJ56_RS17390 |
| NAD-dependent DNA ligase | NJ56_RS13970 |
| DNA mismatch repair protein Vsr | NJ56_RS02495 |
| DNA primase | NJ56_RS11625 |
| DNA-binding protein | NJ56_RS01540 |
| DNA-binding protein | NJ56_RS13980 |
| Replicative DNA helicase | NJ56_RS01450 |
| Mutagenesis and repair protein MucA | NJ56_RS17435 |
| HNH endonuclease | NJ56_RS17615 |
| DNA topoisomerase III | NJ56_RS01485 |
| DNA polymerase V subunit UmuD | NJ56_RS01710 |
| DNA polymerase V subunit UmuC | NJ56_RS17440 |
| Chromosome (Plasmid) partitioning protein ParA | NJ56_RS17460 |
| Methylase | NJ56_RS17225 |
| Replication protein | NJ56_RS17150 |
| DNA-binding protein | NJ56_RS01640 |
| **Transcriptional regulators** |  |
| Repressor protein C2 | NJ56_RS17790 |
| CI repressor | NJ56_RS16805 |
| Putative transcriptional regulator | NJ56_RS17165 |
| Transcriptional regulator | NJ56_RS14800 |
| **Cellular energy homeostasis** |  |
| ATPase AAA | NJ56_RS16020 |
| Adenylate kinase | NJ56_RS14905 |
| ATPase | NJ56_RS17160 |
| ATPase | NJ56_RS02545 |
| ATPase | NJ56_RS01595 |
| **Cell wall polysaccharide biosynthesis** |  |
| Flippase | NJ56_RS04845 |
| Paratose synthase | NJ56_RS04840 |
| CDP-glucose 4,6-dehydratase | NJ56_RS04830 |
| Alpha-D-glucose-1-phosphate cytidylyltransferase | NJ56_RS04825 |
| 4-amino-4-deoxy-L-arabinose-phospho-UDP flippase subunit E | NJ56_RS05080 |
| dTDP-4-dehydrorhamnose 3,5-epimerase | NJ56_RS04835 |
| UDP-glucose 4-epimerase | NJ56_RS04865 |
| Lytic transglycosylase | NJ56_RS01510 |
| Capsular biosynthesis protein | NJ56_RS04870 |
| Glycosyl transferase WbuB | NJ56_RS04880 |
| Glycosyl transferase family 2 | NJ56_RS05075 |
| Tyrosine-protein kinase CpsD | NJ56_RS01440 |
| **Others** |  |
| Beta family protein | NJ56_RS14090 |
| Thymidylate synthase | NJ56_RS14900 |
| Patatin | NJ56_RS14940 |
| Cell division protein | NJ56_RS17795 |
| Protein-disulfide isomerase | NJ56_RS17125 |
| Plasmid stabilization protein | NJ56_RS01635 |
| Secretoglobin family protein | NJ56_RS02525 |
| Lipase | NJ56_RS17580 |
| Putative ATP/GTP binding protein | NJ56_RS01455 |
| Histone-like nucleoid-structuring protein H-NS | NJ56_RS16935 |
| DNA transfer and F pilus assembly protein | NJ56_RS17040 |
| Hypothetical sex pilus assembly and synthesis protein | NJ56_RS17110 |
| Type VI secretion protein | NJ56_RS01585 |
| Lipoprotein | NJ56_RS01565 |
